# Survival outcome following surgical versus non-surgical treatment of colorectal lung metastasis—a retrospective cohort study

**DOI:** 10.1007/s00423-024-03311-1

**Published:** 2024-04-12

**Authors:** Axel Denz, Veronika Hahn, Klaus Weber, Georg F. Weber, Robert Grützmann, Christian Krautz, Maximilian Brunner

**Affiliations:** Department of General and Visceral Surgery, Friedrich-Alexander-University, Krankenhausstraße 12, 91054 Erlangen, Germany

**Keywords:** Colorectal carcinoma, Lung metastases, Surgical resection, Non-surgical treatment, Outcome, Overall survival

## Abstract

**Purpose:**

The optimal management of colorectal lung metastases (CRLM) is still controversial. The aim of this study was to compare surgical and non-surgical treatment for CRLM regarding the prognostic outcome.

**Methods:**

This retrospective single-center cohort study included 418 patients, who were treated from January 2000 to December 2018 at a German University Hospital due to their colorectal carcinoma and had synchronous or metachronous lung metastases. Patients were stratified according the treatment of the CRLM into two groups: surgical resection of CRLM versus no surgical resection of CRLM. The survival from the time of diagnosis of lung metastasis was compared between the groups.

**Results:**

Two- and 5-year overall survival (OS) from the time of diagnosis of lung metastasis was 78.2% and 54.6%, respectively, in our cohort. Patients undergoing pulmonary metastasectomy showed a significantly better 2- and 5-year survival compared to patients with non-surgical treatment (2-year OS: 98.1% vs. 67.9%; 5-year OS: 81.2% vs. 28.8%; *p* < 0.001). Multivariate Cox regression revealed the surgical treatment (HR 4.51 (95% CI = 2.33–8.75, *p* < 0.001) and the absence of other metastases (HR 1.79 (95% CI = 1.05–3.04), *p* = 0.032) as independent prognostic factors in patients with CRLM.

**Conclusion:**

Our data suggest that patients with CRLM, who qualify for surgery, benefit from surgical treatment. Randomized controlled trials are needed to confirm our findings.

**Clinical trial registry number:**

The work has been retrospectively registrated at the German Clinical Trial Registry (DRKS00032938).

**Supplementary Information:**

The online version contains supplementary material available at 10.1007/s00423-024-03311-1.

## Introduction

Colorectal carcinoma (CRC) is the third most common cancer as well as the third leading cause of cancer-related death in Europe and the USA [1]. Due to continuous treatment improvements including multimodal treatment options and improved surgery concepts, CRC is nowadays associated with a good prognosis.

However, about 50% of the patients with CRC had at diagnosis or develop during follow-up a metastatic disease, which significantly impairs the prognosis [2]. Next to the most common location in the liver, colorectal organ metastases also often affect the lung. Colorectal lung metastases (CRLM) occur in approximately 10% of patients with CRC [3, 4]. The 5-year survival of patients with CRLM is currently estimated about 40–60% [5, 6].

While standardized multimodal therapy concepts have been established for liver metastases, the best management for lung metastases remains controversial [7]. This is also reflected in existing guidelines, which usually contain hardly any recommendations for the therapy of lung metastases [8]. There is especially an ongoing discussion, if and when pulmonary metastasectomy is indicated. In an era of increasingly improved chemotherapy and radiotherapy as alternative treatment options, the usefulness of lung metastasis resection is being questioned. This is fueled by the recently published randomized controlled PulMiCC trial, which failed to show a benefit from pulmonary metastasectomy compared to an observational approach [9]. Likewise, a current review and meta-analysis including ten studies could reveal no significant survival difference between the surgical and non-surgical management of CRLM for recently published data and recommended further studies to improve evidence [7].

The aim of this study was to evaluate whether pulmonary metastasectomy of CRLM is associated with a survival benefit for affected patients and to identify patient subgroups that might benefit most from surgical therapy.

## Material and methods

The prospectively maintained Cancer Registry of the Department of Surgery was used to identify patients for this retrospective analysis. All adult patients with colorectal lung metastasis, who were treated for any step of therapy at the department of general and visceral surgery of University Hospital between 01.01.2000 and 31.12.2018, were included to this study. Patients with missing data regarding the therapy of lung metastasis were excluded (*n* = 2). In summary, data from 418 patients with CRLM were analyzed.

Clinical and epidemiological data, treatment, histopathological findings and follow-up data were retrieved partially from the Registry for Colorectal Carcinomas (ERCRC) and partially from the clinical information system. The detailed documentation of the histopathological examinations allowed the classification of all carcinomas in accordance with the 8th edition of the UICC TNM classification [10]. The median follow-up time for the patients was 19 months [range 0–183 months].

This retrospective study was approved by the local ethics committee (23–219-Br). The work has been registrated at the German Clinical Trial Registry (DRKS00032938) and reported in line with the STROCSS criteria [11].

### Treatment of CRLM and follow-up

Management of CRLM was decided for each patient individually by a multidisciplinary tumor board according to the current evidence at the time of treatment.

The surgical approach was chosen according to the location and number of pulmonary nodules. In most patients, pulmonary metastasectomy was performed during one surgery (63 patients, 55%); 33 (29%) and 19 patients (17%) needed two or more than two surgeries. Most patients received open surgery (75%) mostly as segmentectomy (56%) with thoracic lymphadenectomy (65%) (Supp tab. S1). Additional perioperative chemotherapy, radiotherapy or chemoradiotherapy was performed in 40% (46 patients), 7% (eight patients) and 10% (11 patients).

Non-surgical management included the performance of a chemotherapy in 209 patients (69%), of a chemoradiotherapy in 16 (5%), of a radiotherapy in six patients (2%) and an observational non-surgical management in 71 patients (24%). Chemotherapy regimens were selected according to the evidence-based German guideline for colorectal cancer that was valid at the time of treatment and taking into account previous chemotherapies.

All patients were offered a close follow up, beginning quarterly, then semi-annually and finally at least annually. Follow-ups contained physical examination, determination of carcinoembryonic antigen (CEA) levels and preferable a chest and abdomen CT scan or an abdominal ultrasonography and chest X-ray.

### Statistical analysis

SPSS® Version 28 (IBM, Armonk, NY) was used to analyze the data. Comparisons of metric and ordinal data were calculated with the Student *t* test or Mann–Whitney *U* test. The Chi-square test was used for categorical data. Overall survival (OS) was calculated for the period between the date of diagnosis of CRLM and the date of death or last follow-up. Possible factors related to the overall (OS) of patients were tested using univariate and multivariate analysis. Variables with a *p* ≤ 0.05 in univariate analysis were used for multivariate analysis by Cox regression model. Survival curves were plotted using the Kaplan–Meier method and compared with the log-rank test. A *p* value ≤ 0.05 was considered statistically significant.

## Results

### Dataset of patients

A total of 418 patients met the inclusion criteria and were analyzed. One hundred fifteen patients underwent a surgical therapy of their CRLM, and 303 received a non-surgical management of their CRLM.

All patients with surgical therapy received R0 resection. Of the patients with additional thoracic lymphadenectomy during pulmonary metastectomy (75 patients), 21% had histologically proven thoracic lymph node metastasis (Supp. tab. S1).

### Patient characteristics

Baseline characteristics of the included patients at the time of first diagnosis and about the primary tumor are presented in Table 1. Significant differences in baseline characteristics contained a lower age (61 vs. 62 years, *p* = 0.036), a higher proportion of rectal carcinoma (71 vs. 54%, *p* = 0.003) and less synchronous metastasis (40 vs 54%, *p* = 0.012) in the surgical group (Table 1).

**Table 1:** Baseline characteristics of patients with colorectal lung metastases

	All patients	Patients with surgical resection of lung metastases	Patients without surgical resection of lung metastases	*p* value
Number	418	115	303	
Age at first diagnosis (years), median (IQR)	62 (15)	61 (14)	62 (17)	**0.036**
Gender, *n* (%)				0.432
Female	163 (39)	41 (36)	122 (40)	
Male	255 (61)	74 (64)	181 (60)	
BMI (kg/m^2^), median (IQR)	26.1 (6.0)	27.1 (5.6)	25.8 (6.1)	0.081
Primary tumor location, *n* (%)				**0.003**
Colon	160 (38)	29 (25)	131 (43)	
Rectum	246 (59)	83 (71)	164 (54)	
Synchronous colon and rectum	12 (3)	4 (4)	8 (3)	
CEA at first diagnosis (ng/ml), median (IQR)	9.5 (37.5)	6.2 (23.5)	13.2 (109.8)	0.257
Primary T category, *n* (%)				0.056
(y)pT0	3 (1)	1 (1)	2 (1)	
(y)pT1	5 (1)	1 (1)	4 (1)	
(y)pT2	58 (14)	21 (18)	37 (12)	
(y)pT3	237 (57)	73 (64)	164 (54)	
(y)pT4	96 (23)	16 (14)	80 (26)	
Unknown	19 (5)	3 (3)	16 (5)	
Primary *N* category, *n* (%)				0.152
(y)pN0	137 (34)	46 (40)	91 (30)	
(y)pN1	149 (37)	41 (36)	108 (36)	
(y)pN2	115 (29)	25 (22)	90 (30)	
Unknown	17 (4)	3 (3)	14 (5)	
Primary M category, *n* (%)				**0.012**
cM0	208 (50)	69 (60)	139 (46)	
cM1	210 (50)	46 (40)	164 (54)	
Primary metastases location,* n* (%)				** < 0.001**
Only liver	63 (30)	11 (24)	52 (32)	
Only lung	30 (14)	16 (35)	14 (9)	
Liver and lung	59 (28)	12 (26)	47 (29)	
Only peritoneum	11 (5)	2 (4)	9 (6)	
Others (incl. multiple)	46 (22)	5 (11)	42 (26)	
Primary grading, *n* (%)				0.581
G1	12 (3)	3 (3)	9 (3)	
G2	229 (55)	68 (59)	161 (53)	
G3	75 (18)	16 (14)	59 (20)	
Unknown	102 (24)	28 (24)	74 (24)	

*BMI* body mass index

Bolded *p*-values indicate significant *p*-values

### Lung metastases specific characteristics

Lung metastases occurred in median 16 months after first colorectal carcinoma diagnosis and were mostly metachronous (71%), bilateral (65%) and multiple (70%). Patients with a surgical therapy of CRLM were significantly more likely to have a unilateral (65 vs. 23%, *p* < 0.001) and singular (44 vs. 16%, *p* < 0.001) pattern of CRLM, had a significantly lower CEA values (2.7 vs. 10.2 ng/ml, *p* < 0.001) and showed significantly fewer other existing metastases (27 vs. 73%, *p* < 0.001). Chemotherapy was performed more often in the non-surgical patient group (72 vs 50%, *p* < 0.001) (Table 2).

**Table 2:** Lung metastases specific characteristics of patients with colorectal lung metastases

	All patients (*n* = 418)	Patients with surgical resection of lung metastases (*n* = 115)	Patients without surgical resection of lung metastases (*n* = 303)	*p* value
Age at diagnosis of LM (years), median (IQR)	64 (16)	63 (16)	65 (16)	0.054
Type of LM, *n* (%)				0.188
Synchronous	122 (29)	28 (24)	94 (31)	
Metachronous	296 (71)	87 (76)	209 (69)	
Time between first diagnosis to diagnosis of LM (months), median (IQR)	16 (37)	19 (37)	15 (37)	0.250
Location of LM, *n* (%)				** < 0.001**
Unilateral	145 (35)	75 (65)	70 (23)	
Bilateral	273 (65)	40 (35)	233 (77)	
Number of LM, *n* (%)				** < 0.001**
1	100 (24)	51 (44)	49 (16)	
2–5	137 (33)	51 (44)	86 (28)	
6–10	53 (13)	5 (4)	48 (16)	
> 10	105 (25)	3 (3)	102 (34)	
Unknown	23 (6)	5 (4)	18 (6)	
CEA at first diagnosis of LM (ng/ml), median (IQR) (*n* = 166)	7.9 (31.2)	2.7 (12.5)	10.2 (47.0)	** < 0.001**
Tumor situation at diagnosis of LM, *n* (%)				** < 0.001**
No previous/existing other metastasis*	116 (28)	57 (50)	59 (20)	
Previous metastasis, now R0*	49 (12)	27 (24)	22 (7)	
Existing other metastasis*	253 (61)	31 (27)	222 (73)	
Chemotherapy for LM performed, *n* (%)	256 (66)	57 (50)	199 (72)	** < 0.001**

^*^Except for lung metastasis

Bolded *p*-values indicate significant *p*-values

### Prognostic factors for overall survival

**Fig. 1:**
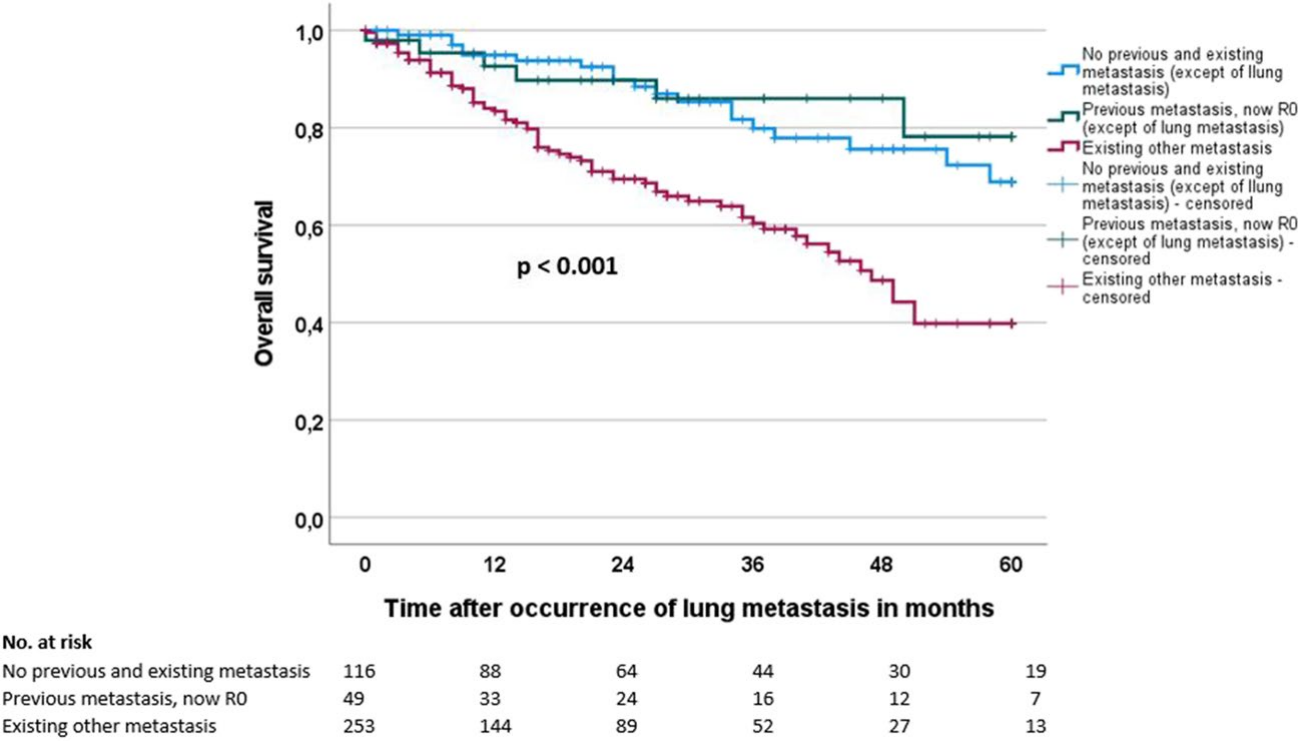
Overall survival of patients with colorectal lung metastasis according to tumor situation at diagnosis of lung metastasis (no previous metastasis vs. previous metastasis, now R0 vs. existing other metastasis)

**Fig. 2:**
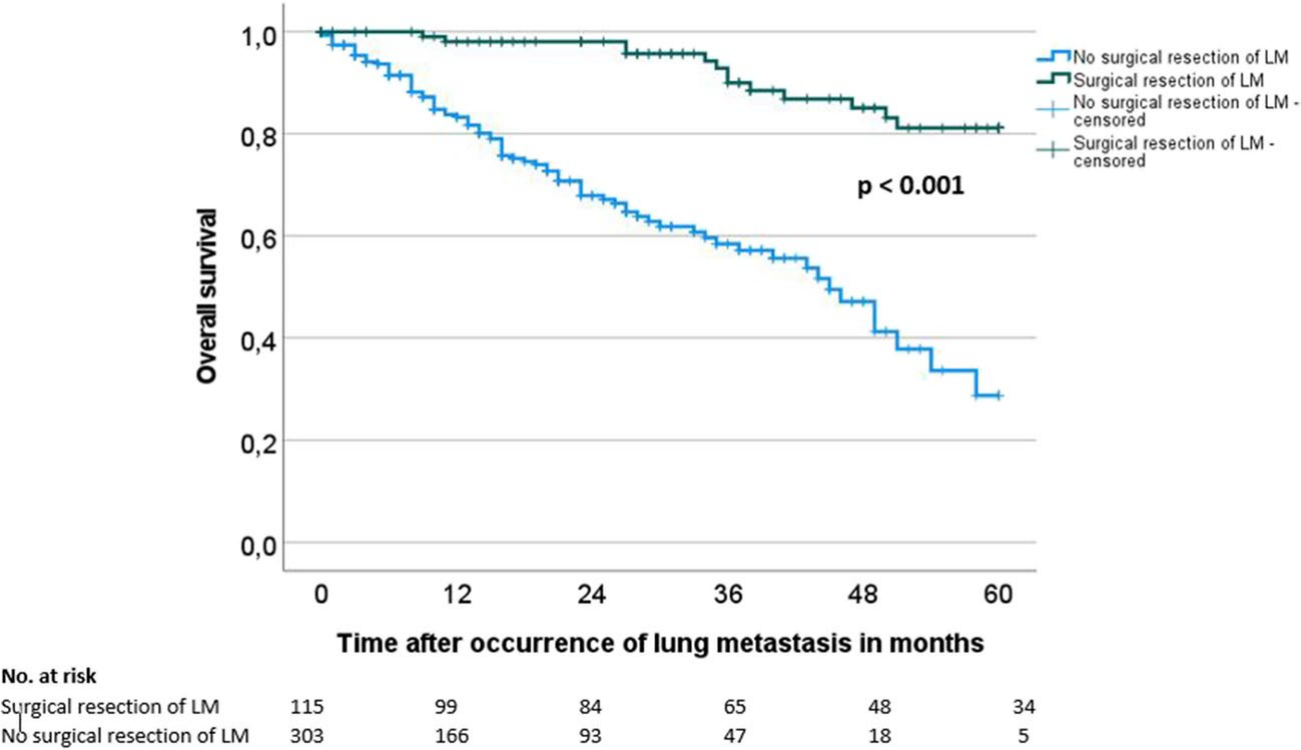
Overall survival of patients with colorectal lung metastasis according to therapy performed (surgical vs. non-surgical therapy)

Two- and 5-year overall survival (OS) from the time of diagnosis of lung metastasis was 78.2% and 54.6%, respectively, in our cohort. Univariate analysis indicated that the location of CRLM (uni- vs. bilateral, *p* = 0.005), the number of CRLM (p < 0.001), the tumor situation at diagnosis of CRLM (no previous and existing metastasis vs. previous metastasis, now R0 vs. existing other metastasis, *p* < 0.001; Fig. 1) and the therapy approach (surgical vs. non-surgical, *p* < 0.001; Fig. 2) significantly influence the overall survival after diagnosis of CRLM (Table 3).

**Table 3:** Prognostic factors of patients with colorectal lung metastases for overall survival (OS)

	*n*	Overall survival (OS) from time of occurrence of LM					
		Univariate			Multivariate		
		2-year OS/SE (%)	5-year OS/SE (%)	*p*	HR	95% CI	*p* value
Age at first tumor diagnosis				0.159			
≤ 62 years*	219	80.7 / 3.2	56.4 / 5.5		-	-	-
> 62 years*	199	75.6 / 3.7	53.2 / 6.2				
Gender				0.438			
Female	163	81.5 / 3.8	55.6 / 6.9		-	-	-
Male	255	76.3 / 3.1	53.8 / 5.2				
Primary tumor location				0.118			
Colon	160	70.7 / 4.5	53.0 / 7.2				
Rectum	246	83.3 / 2.8	55.6 / 5.2		-	-	-
Synchronous colon and rectum	12	59.3 / 18.5	59.3 / 18.5				
Primary T category (*n* = 399)				0.868			
(y)pT0/1/2	66	73.3 / 6.5	57.7 / 8.1		-	-	-
(y)pT3/4	333	79.8 / 2.6	53.9 / 4.9				
Primary N category (*n* = 401)				0.698			
(y)pN0	137	79.0 / 4.2	52.4 / 8.1		-	-	-
(y)pN +	264	78.0 / 3.1	55.4 / 5.0				
Primary M category				0.519			
cM0	208	77.7 / 3.5	59.9 / 5.5		-	-	-
pM1	210	78.7 / 3.3	48.2 / 6.2				
Primary differentiation (*n* = 316)				0.513			
G1/G2	241	79.2 / 3.1	54.8 / 5.6		-	-	-
G3	75	82.7 / 5.0	62.9 / 9.2				
Age at diagnosis of LM				0.215			
≤ 64 years*	211	80.8 / 3.1	55.1 / 5.5		-	-	-
> 64 years*	207	75.4 / 3.7	55.7 / 5.9				
Type of LM				0.621			
Synchronous	122	78.6 / 4.3	56.1 / 7.3		-	-	-
Metachronous	296	78.1 / 2.9	53.7 / 5.0				
Time between first diagnosis to diagnosis of LM				0.161			
≤ 16 months	213	81.1 / 3.1	56.8 / 5.7		-	-	-
> 16 months	205	74.7 / 3.8	51.9 / 6.0				
Location of LM				**0.005**			
Unilateral	145	86.9 / 3.2	66.6 / 5.7		1.00		
Bilateral	273	72.9 / 3.3	45.6 / 5.9		1.19	0.56 – 2.52	0.659
Number of LM (*n* = 395)				** < 0.001**			
1	100	83.6 / 4.2	70.5 / 6.6		1.00		
2–5	137	86.0 / 3.5	57.0 / 6.7		1.27	0.59 – 2.76	0.546
6–10	53	64.0 / 7.7	52.8 / 9.6		1.86	0.69 – 5.06	0.223
> 10	105	69.5 / 5.8	20.6 / 11.0		1.42	0.55 – 3.67	0.475
CEA at diagnosis of LM (*n* = 166)				0.085			
≤ 7.9 ng/ml*	83	79.2 / 4.8	53.2 / 7.4		-	-	**-**
> 7.9 ng/ml*	83	65.6 / 6.3	38.0 / 8.6				
Tumor situation at diagnosis of LM				** < 0.001**			
No previous and existing metastasis**	116	89.8 / 3.2	68.9 / 6.7		1.00		
Previous metastasis, now R0**	49	89.8 / 4.9	78.2 / 9.2		1.05	0.42 – 2.66	0.915
Existing other metastasis**	253	69.5 / 3.6	39.8 / 5.9		1.79	1.05 – 3.04	**0.032**
Chemotherapy (*n* = 391)				0.229			
Yes	256	77.8 / 2.9	47.1 / 5.3		-	-	-
No	135	79.5 / 4.2	72.1 / 5.6				
Surgical resection of LM				** < 0.001**			
Yes	115	98.1 / 1.3	81.2 / 4.9		1.00		
No	303	67.9 / 3.4	28.8 / 7.5		4.51	2.33 – 8.75	** < 0.001**

*LM* lung metastasis

^*^Cutoff = median

^*^^*^Except for lung metastasis

Bolded *p*-values indicate significant *p*-values

Multivariate analysis revealed that a surgical resection of CRLM (HR 4.51 (95% CI = 2.33–8.75, *p* < 0.001) and the absence of other metastases (HR 1.79 (95% CI = 1.05–3.04), *p* = 0.032) were significant independent prognostic factors regarding the OS (Table 3).

### Subgroup analysis

Subgroup analysis of all subgroups identified as prognostic factors in univariate analysis (location of CRLM, number of CRLM, tumor situation at diagnosis of CRLM) showed that patients with unilateral (*p* < 0.001) as well as bilateral CRLM (*p* < 0.001), with one (*p* = 0.003) as well as two to five CRLM (*p* < 0.001) and with no previous and existing other metastasis (*p* < 0.001) as well as with previous, but R0-resected metastasis (*p* = 0.027) as well as with existing other metastasis (*p* < 0.001) benefit from surgical therapy of CRLM. Only in the group with more than six CRLM surgical resection of CRLM reached no significance (*p* = 0.144). Again, surgical therapy of CRLM was associated with a significant better overall survival in still more selected subgroups (one CRLM and no other metastasis; ≥ 2 CRLM and existing other metastasis; only patients with combined liver and lung metastasis) (Supp. tab. S2). Surgical details as well as preoperative suspicion and histologically proven existence of thoracic lymph node metastasis did not affect the overall survival in the surgical subgroup (Supp. tab. 1).

## Discussion

The optimal therapy of CRLM remains an ongoing controversial topic, especially against the background of increasingly improved chemotherapeutic and radiotherapeutic therapy options. The current data situation on this topic is divergent, which justifies the need for further evidence [7].

The 2- and 5-year overall survival (OS) from the time of diagnosis of lung metastasis was 78.2% and 54.6%, respectively, in our cohort, which is similar to reported survival rates in the literature [5, 6, 12].

Our data suggest that the surgical therapy of CRLM is associated with a significant survival advantage for patients with CRLM compared to a non-surgical treatment in a selected patient cohort (3). These results are in line with several other study results, which show an approximately 40–50% better 5-year survival following pulmonary metastectomy for CRLM [5, 13–18]. In contrast, there are some studies, which revealed comparable survival outcomes in surgical and non-surgical treated patients [9, 19, 20]. These results mainly concern especially the more recent ones and are explained by the advent of novel chemotherapeutic regimes and interventional therapy options. However, if we only include the more recent data (from 2010) in our collective, the significant survival benefit of the surgical therapy of CRLM remains.

An important aspect regarding these data, which allows the survival advantage in the surgical group to be interpreted with caution, may be a significant selection bias favoring the surgical group. Comparing the baseline and lung metastasis specific characteristics between the surgical and the non-surgical group, those selected for pulmonary metastasectomy more frequently had prognostically favorable characteristics (e.g. lower metastatic disease rate at first diagnosis, less bilateral pattern and lower number of CRLM, lower rate of existing other metastasis next to CRLM). However, in multivariate analysis including these mentioned divergent parameters between the groups pulmonary metastasectomy remain an independent highly significant prognostic parameter. Moreover, we performed a subgroup analysis of all identified significant prognostic factors (location of CRLM, number of CRLM, tumor situation at diagnosis of CRLM) to reduce the influence of potential selection bias and to identify subgroups that might benefit most from surgical therapy. With the exception of patients with more than six CRLM, surgical therapy was associated with a significant survival advantage in all subgroups formed. Combinations of two prognostic factors reflecting low and high tumor burden (one CRLM and no other metastasis; ≥ 2 CRLM and existing other metastasis) also led to a significant prognostic benefit of surgical therapy of CRLM. In all subgroup analysis the percentage of surgically treated patients was higher in subgroups with less tumor burden reflecting again a selection bias, which limits the validity of the data. However, our data suggest that with a rational selection like in our collective also patients with high tumor burden can benefit from pulmonary metastasectomy. One factor that may be underrepresented in our analysis, because it cannot be determined with sufficient validity from the available data, is the general condition of the patient when deciding whether or not to treat the lung metastases surgically.

Not investigated in our study, but another important aspect to consider in the decision between surgical and non-surgical therapy for colorectal lung metastases is the impact on quality of life. Patients who undergo surgery may experience periods of chemotherapy-free time, which may improve their quality of life. However, it is important to acknowledge that surgery carries the risk of postoperative morbidity, which can negatively affect both quality of life and even survival [21].

Next to surgical resection of CRLM we identified the presence of existing other metastasis at diagnosis of CRLM as independent prognostic parameter in multivariate analysis. Patients with previous, but now not anymore existing metastases, had a similar survival as patients without any previous or existing metastases underlining the importance and usefulness of effective metastasis treatment in colorectal carcinoma.

Additionally, in the univariate analysis, the location of colorectal lung metastases (unilateral vs. bilateral) as well as the number of metastases had an impact on overall survival. These findings are consistent with previous studies that have described the predictive influence of these factors on patient outcomes [12].

The present study has several limitations. First, it is a single-center study which has the advantage of a homogeneous therapy concept, but makes it difficult to generalize the results. Second, the retrospective analysis of prospective recorded data may have incurred some bias. Third, the number of patients is collected over a long period of 18 years. Therapy options improved over the years especially concerning adjuvant chemotherapy. However, there was no significant difference of the results between earlier und the later years of data period. Fourth, retrospective data about this topic are always influenced by a selection bias, which has already been discussed in detail above. Randomized controlled trials are needed to overcome this limitation.

## Conclusion

The present study confirms the importance of surgical therapy of CRLM, as it is associated with a significant better overall survival in a selected patient cohort—even in patients with high tumor burden. Randomized controlled trials are needed to clarify this important question in a patient collective without selection bias.

### Supplementary Information

Below is the link to the electronic supplementary material.

Supplementary file1 (DOCX 21 KB)

## Data Availability

All data generated or analyzed during this study are included in this published article. The publication contains one supplemental table.

## References

[1] Siegel RL, Miller KD, Fuchs HE, Jemal A (2022). Cancer statistics, 2022. CA Cancer J Clin.

[2] Labianca R, Beretta GD, Kildani B, Milesi L, Merlin F, Mosconi S (2010). Colon cancer. Crit Rev Oncol Hematol.

[3] Kim HK, Cho JH, Lee HY, Lee J, Kim J (2014). Pulmonary metastectomy for colorectal cancer: how many nodules, how many times?. World j Gastroenterol.

[4] Fiorentino F, Hunt I, Teoh K, Treasure T, Utley M (2010). Pulmonary metastasectomy in colorectal cancer: a systematic review and quantitative synthesis. J R Soc Med.

[5] Gonzalez M, Poncet A, Combescure C, Robert J, Ris HB, Gervaz P (2013). Risk factors for survival after lung metastasectomy in colorectal cancer patients: a systematic review and meta-analysis. Ann Surg Oncol.

[6] Schirren J, Schirren M, Lampl L, Editorial SS (2017). Surgery for pulmonary metastases: quo vadis?. Eur J Cardiothorac Surg.

[7] Ratnayake CBB, Wells CI, Atherton P, Hammond JS, White S, French JJ, Manas D, Pandanaboyana S (2021). Meta-analysis of survival outcomes following surgical and non surgical treatments for colorectal cancer metastasis to the lung. ANZ J Surg.

[8] Leitlinienprogramm Onkologie (Deutsche Krebsgesellschaft, Deutsche Krebshilfe, AWMF): S3-Leitlinie Kolorektales Karzinom, Langversion 2.1, 2019, AWMF Registrierungsnummer: 021/007OL, http://www.leitlinienprogrammonkologie.de/leitlinien/kolorektales-karzinom/. Accessed 18 Jan 2023

[9] Treasure T, Farewell V, Macbeth F, Monson K, Williams NR, Brew-Graves C, Lees B, Grigg O, Fallowfield L, PulMiCC Trial Group (2019) Pulmonary Metastasectomy versus Continued Active Monitoring in Colorectal Cancer (PulMiCC): a multicentre randomised clinical trial. Trials 20(1):718. 10.1186/s13063-019-3837-y 10.1186/s13063-019-3837-y PMC6909580 31831062

[10] UICC (International Union of Cancer) (2017) TNM Classifcation of malignant tumours 8th edn, Brierley JD, Gospodarowicz MK, Wittekind, Ch. (eds). Wiley Blackwell, Oxford

[11] Mathew G, Agha R, for the STROCSS Group (2021). STROCSS 2021: Strengthening the Reporting of cohort, cross-sectional and case-control studies in Surgery. Int J Surg.

[12] Dudek W, Schreiner W, Hohenberger W, Klein P, Sirbu H (2017). Forty-two years' experience with pulmonary resections of metastases from colorectal cancer. Thorac Cardiovasc Surg.

[13] Inoue M, Ohta M, Iuchi K (2004). Benefits of surgery for patients with pulmonary metastases from colorectal carcinoma. Ann Thorac Surg.

[14] McCormack PM, Burt ME, Bains MS (1992). Lung resection for colorectal metastases: 10-year results. Arch Surg.

[15] Robinson BJ, Rice TW, Strong SA, Rybicki LA, Blackstone EH (1999). Is resection of pulmonary and hepatic metastases warranted in patients with colorectal cancer?. J Thorac Cardiovasc Surg.

[16] Limmer S, Oevermann E, Killaitis C, Kujath P, Hoffmann M, Bruch HP (2010). Sequential surgical resection of hepatic and pulmonary metastases from colorectal cancer. Langenbecks Arch Surg.

[17] Wiegering A, Riegel J, Wagner J (2017). The impact of pulmonary metastasectomy in patients with previously resected colorectal cancer liver metastases. PLoS ONE.

[18] Tampellini M, Ottone A, Bellini E (2012). The role of lung metastasis resection in improving outcome of colorectal cancer patients: results from a large retrospective study. Oncologist.

[19] Meimarakis G, Angele M, Conrad C (2013). Combined resection of colorectal hepatic–pulmonary metastases shows improved outcome over chemotherapy alone. Langenbecks Arch Surg.

[20] Boysen AK, Spindler K, Høyer M (2018). Metastasis directed therapy for liver and lung metastases from colorectal cancer: a population-based study. Int J Cancer.

[21] Beck C, Weber K, Brunner M, Agaimy A, Semrau S, Grützmann R, Schellerer V, Merkel S (2020). The influence of postoperative complications on long-term prognosis in patients with colorectal carcinoma. Int J Colorectal Dis.

