# Supplementary material for: Survival outcome following surgical versus non-surgical treatment of colorectal lung metastasis—a retrospective cohort study
Source: Langenbecks Arch Surg. 2024 Apr 12;409(1):121. doi: 10.1007/s00423-024-03311-1 (PMC11009744; doi:10.1007/s00423-024-03311-1)
Supplement: Supplementary file 1 — Supplementary file1 (DOCX 21 KB) [file 423_2024_3311_MOESM1_ESM.docx]

**Supp. tab. S1:** Details of lung metastasis surgery

|  | **All patients**  **(n = 115)** | **Overall survival from time of occurence of LM** | | |
| --- | --- | --- | --- | --- |
|  |  | **2-year-OS/SE (%)** | **5-year-OS/SE (%)** | **p** |
| **Number of pulmonary metastectomy**  **1**  **2**  **> 2** | 63 (55)  33 (29)  19 (17) | 96.2 / 2.6  100 / -  100 / - | 72.2 / 8.5  80.6 / 8.9  100 / - | 0.088 |
| **Surgical approach**  **Thoracotomy**  **Thoracoscopy** | 86 (75)  28 (25) | 98.4 / 1.6  100 / - | 86.5 / 5.2  66.2 / 13.4 | 0.088 |
| **Extent of resection**  **Wedge resection**  **Segmentectomy** | 51 (44)  66 (56) | 100 / -  97.9 / 2.1 | 77.3 / 9.3  83.5 / 6.3 | 0.654 |
| **Additional thoracic lymphadenectomy**  **Yes**  **No** | 75 (65)  40 (35) | 98.1 / 1.8  100 / - | 87.7 / 5.3  66.8 / 11.8 | 0.077 |
| **Resection margin**  **R0**  **R1** | 115 (100)  0 (0) | - | - | - |
| **Thoracic lymph node metastasis (n = 75)**  **Yes**  **No** | 16 (21)  59 (79) | 90.0 / 9.5  100 / - | 77.1 / 14.4  89.7 / 5.7 | 0.382 |
| **Preoperative suspicious thoracic lymph nodes**  **Yes**  **No** | 13 (11)  102 (89) | 100 / -  98.7 / 1.3 | 88.9 / 10.5  80.7 / 5.6 | 0.862 |

LM = lung metastasis.

**Supp. tab. S2:** Impact of surgical vs. non-surgical therapy on overall survival (OS) for subgroups of patients with colorectal lung metastasis

|  |  |  |  | **Overall survival (OS) from time of occurrence of LM** | | |
| --- | --- | --- | --- | --- | --- | --- |
|  |  | **Surgical resection of LM** | **n** | **2-year-OS/SE (%)** | **5-year-OS/SE (%)** | **p** |
| **Location of LM** | **Unilateral** | **No**  **Yes** | 70  75 | 73.8 / 6.4  96.9 / 2.1 | 51.5 / 10.6  77.2 / 6.6 | **< 0.001** |
|  | **Bilateral** | **No**  **Yes** | 233  40 | 65.9 / 4.0  100 / - | 22.4 / 8.3  87.6 / 6.8 | **< 0.001** |
| **Number of LM** | **1** | **No**  **Yes** | 49  51 | 69.1 / 8.0  95.5 / 3.1 | 50.7 / 13.5  83.4 / 7.3 | **0.003** |
|  | **2-5** | **No**  **Yes** | 86  51 | 73.1 / 6.2  100 / - | 22.2 / 11.7  80.6 / 7.3 | **< 0.001** |
|  | **> 6** | **No**  **Yes** | 150  8 | 65.3 / 4.9  100 / - | 21.7 / 11.3  60.0 / 21.9 | 0.144 |
| **Tumor situation at diagnosis of LM** | **No previous and existing metastasis*** | **No**  **Yes** | 59  57 | 79.0 / 6.2  98.1 / 1.9 | 32.6 / 16.0  85.9 / 6.0 | **< 0.001** |
|  | **Previous metastasis, now R0*** | **No**  **Yes** | 22  27 | 78.3 / 11.7  96.0 / 3.9 | 62.7 / 16.9  86.4 / 9.8 | **0.027** |
|  | **Existing other metastasis*** | **No**  **Yes** | 222  31 | 63.7 / 4.1  100 / - | 25.0 / 8.2  71.9 / 9.9 | **< 0.001** |
| **Selected subgroups** | **1 LM, no other metastasis** | **No**  **Yes** | 16  44 | 75.0 /12.8  94.8 / 3.6 | 40.0 / 19.8  88.0 / 7.3 | **0.001** |
|  | **≥ 2 LM, existing other metastasis** | **No**  **Yes** | 176  22 | 64.1 / 4.6  100 / - | 18.0 / 8.4  69.4 / 12.9 | **< 0.001** |
|  | **Only patients with previous or current liver and lung metastasis** | **No**  **Yes** | 122  37 | 69.2 / 5.4  97.0 / 3.0 | 33.0 / 15.7  81.5 / 8.7 | **< 0.001** |

LM = lung metastasis; * except of lung metastasis.
